# Practice of Environmentally Significant Behaviours in Rural China: From Being Motivated by Economic Gains to Being Motivated by Environmental Considerations

**DOI:** 10.3390/bs7030059

**Published:** 2017-08-22

**Authors:** Yanyan Chen

**Affiliations:** Graduate School of Culture and Information Science, Doshisha University, Kyoto 610-0394, Japan; yachen@mail.doshisha.ac.jp

**Keywords:** environmentally motivated behaviours, norm-activation theory, demographic factors, categorical data analysis, social survey

## Abstract

A continuous and increasing crisis that present-day China is facing is environmental degradation. The cultivation of citizens who have environmentally friendly behaviours has been deemed as a fundamental way to solve environmental crises. However, the main focus of environmentalism studies has been urban residents, whereas rare research attention was put on rural Chinese. This paper focuses on environmentally significant behaviours in rural China and aims to clarify the practice of five environmentally significant behaviours and two motivations underlying these behaviours. In total, 508 rural residents in 51 villages of Ningyang county were interviewed. Analytical results derived from survey data showed that environmentally significant behaviours are widely conducted in rural areas. However, these behaviours are mainly motivated by economic gains rather than environmental considerations. In addition, based on the norm-activation theory and considering the influences of demographic factors, the formation of environmentally motivated behaviours were quantitatively analysed. Analytical results indicated that the more people worried about environmental deterioration, the more likely they were to form environmentally motivated behaviours, and people who ascribe the most important environmental responsibility to the government are less likely to form environmentally motivated behaviours. Increasing people’s anxiety towards the environment, decreasing people’s dependency on the government in protecting the environment, and using females, the elderly, and people with low income and education levels as the main targets of environmental education are suggested to promote environmentally motivated behaviours in rural China.

## 1. Introduction

Environmental impacts have largely been a by-product of human desires for physical comfort, personal security, and so forth [1]. Remarkable economic growth and industrialization contributed significantly to people’s welfare but also created increasing serious environmental degradation. Environmental problems are described as crises of people’s values [2,3] and maladaptive behaviours [4]. From this perspective, the cultivation of citizens who have an environmentally friendly consciousness and responsible behaviours is particularly important when seeking to solve current environmental problems.

Rural China has a distinctive society compared to cities in China. The long-time institutional, economic, and social segmentations make rural China a different, yet coexisting system with the cities in China [5,6,7]. Environmentally significant behaviours are derived from the interaction among different attitudes and behaviours toward the environment in a specific society or community [8]. Therefore, individuals embedded in the rural background of China are supposed to have distinctive behavioural patterns compared to urban China. However, the main focus of environmentalism studies has been urban residents. Regarding study objectives, question design and the description of the system are, for the time being, more suitable for urban residents in the developed areas of China [9]. In 2003, the Chinese General Social Survey (CGSS) was launched to gather longitudinal data on social trends and the social structure. Whereas CGSS 2003 gave a sense of the Chinese people’s environmental attitudes, its scope was limited to urban samples [6]. As with global trends, present-day China is experiencing rapid urbanization. Material life of rural residents has been greatly enriched, and rural consumption is rising remarkably [10,11,12]. However, rapid economic growth has also created increasingly serious environmental problems in rural areas.

Based on the above background, this paper focused on analysing the environmentally significant behaviours in rural China and aimed at answering the following research questions: (1) to which extent rural residents are conducting environmentally significant behaviours in their daily life and what is the underlying motivation? (2) What are the influential factors of environmentally significant behaviours and how can we improve these behaviours in rural China?

## 2. Five Environmentally Significant Behaviours and Two Motivations

Two dominant perspectives have been used to study environmentally significant behaviours: one focused on impact and a second focused on intention [1,13]. Environmentally significant behaviours can be defined by their impacts and the extent they change the availability of materials or alter the structure and dynamics of ecosystems, or they can be defined from the actor’s standpoint as behaviours that are undertaken with the intention of benefiting the environment [1]. In this paper, behaviours that actually contribute to the sustainability of the environment were included and the motivations underlining these behaviours were also considered.

Distinctive types of environmentally significant behaviours have been discussed in previous research [1,14,15,16]. These types can be generally divided into environmental citizenship behaviours (such as signing an environmental petition, or belonging to an environmental group) or environmentally significant behaviours in the private sphere (such as purchasing environmentally friendly household goods and services, recycling, or reusing). In this paper, environmentally significant behaviours in the private sphere were focused. In daily life, there are many behaviours that influence the environment. Although the environmental impact of any individual’s personal behaviour is small, a significantly huge impact will emerge if many people independently engage in the same behaviours in daily life. This paper focuses on rural residents’ environmentally significant behaviours in the private sphere and tries to clarify the practicing status of the following five behaviours in rural areas: (1) the purchase of eco-friendly products; (2) reuse or recycling; (3) water saving; (4) energy saving; and (5) the use of one’s own shopping bag.

Motivation is defined as the driving force behind the behaviours that leads individuals to pursue some things [17]. Axelrod (1994) [18] identified a tribrach classification of motivational domains underlying people’s behaviours: (a) economic motivation, which refers primarily to goals such as economic security, material rewards, or the avoidance of economic, material, or time costs; (b) social motivation, which indicates that seeking belongingness and acceptance from others is a central guiding force in decisions to act; and (c) universal motivation, which involves the pursuit of self-respect from making a contribution to the betterment of the world. Stern, Dietz, and Kalof (1993) [19] presumed that environmentally significant behaviours derive from any of three value orientations: egoistic, social-altruistic, or biospheric. These identifications of diverse motivational domains deepened our understanding regarding the motivations that cause human behaviours. However, these classifications are too general to serve as procedural guidelines to behavioural interventions or motivational campaigns. In this paper, analysis objects were specified to two motivations that may cause people’s environmentally significant behaviours: (1) economic motivation, which refers to the motivation of pursuing economic gains; and (2) environmental motivation, which refers to the motivation of benefiting the environment.

It is likely that there would be multiple motivations implicated in any given behaviour. The performance of environmentally significant behaviours in daily life may be derived from personal environmental intent but also may be a matter of household routine or from an economic purpose, or a combination of several motivations. However, the main motivation that played the decisive role in causing these behaviours for various people is likely to be different. Individuals with a greater extent of environmental consciousness are more likely to conduct the behaviours in consideration of the environment, whereas individuals with less environmental consciousness may be more likely to do so because of other reasons, such as economic gains. This paper placed individuals into an either-or situation, to benefit the environment or pursue economic gains, to rethink their behaviours in daily life and make their choices.

The purposes of this paper are to clarify the practice of the proposed five environmentally significant behaviours in rural areas of China and to explore the specific motivation that underlies these behaviours. Additionally, this paper attempts to examine the formation of behaviours that are motivated by an environmental consideration to supply some clues as to how to promote environmentally significant behaviours in rural areas.

## 3. Theories and Hypotheses

Economic motivation is able to initiate environmentally significant behaviours; however, it was argued that it is unable to produce durable behavioural change. When economic incentives terminate, environmentally significant behaviours are difficult to maintain [20,21]. Economic rewards were shown to have a significant negative effect on intrinsic motivation in conducting various behaviours [22]. In present-day China, rural areas are experiencing fast economic growth. Environmentally significant behaviours stimulated by economic gains are very likely to fade when economic factors are no longer taken as a decisive element in their decision making. Therefore, the shift in environmentally significant behaviours from being motivated by economic gains to environmental considerations is particularly important in the long-term sustainability of rural China.

Environmental motivation has always been discussed in the context of altruism because “the accomplishments of such movements benefit most members of a society and the benefits accrue to an individual whether or not he or she actively participates in the movement” [23]. The model of self-interest theory supplies little explanation for this type of motivation, whereas the norm-activation theory proposed by Schwartz (1970, 1977) [24,25] is assumed to be helpful in explaining the formation of environmental motivation. This theory was originally proposed to explain “helping behaviour”; however, it has been extended extensively to apply to environmental behaviours [23,26,27]. According to this theory, people’s awareness of consequences (AC) and the ascription of responsibility (AR) are the two main factors in activating people’s moral obligation and causing altruistic behaviour. Environmental anxiety is generated from the evaluation of environmental consequence and indicates people’s worries about the environment [27]. This paper takes people’s environmental anxiety as a measurement of AC and proposes that the more people worry about environmental deterioration, the more likely they will be environmentally motivated to conduct these behaviours. Governments, corporations, and citizens are entities that can reasonably be ascribed responsibility for the environment. The ascription of (ecological) responsibility to powerful others (such as God or the government) leads to a lack of motivation among people to conduct the behaviour [28,29]. Citizens who exert their influences on the environment in their various roles as consumers, voters, and tax payers are both the victims and villains of environmental deterioration. Whether they recognize their responsibilities in protecting the environment is supposed to affect the formation of their environmentally motivated behaviours. This paper takes people’s ascription of environmental responsibility as a measurement of AR and proposes that the more people ascribe the most important environmental responsibility to citizens, the more likely they are to form environmentally motivated behaviours.

The influences of demographic factors on people’s environmentally significant behaviours have been subjected to substantial empirical study. Females have been deemed as more environmentally friendly than males due to their biospheric orientation [19] and traditional roles as caregivers, nurturers, mothers, and protectors of children [6]. Young generations were argued to be more concerned with the environment because they were less integrated into the dominant social order that is deemed as the root cause of environmental problems, and are more open to new ideas [30,31,32,33]. Social class, which is generally indicated by education, income, or occupational prestige, was also proved to be positively related to people’s environmental consciousness and behaviours [33] because once the basic physical needs are satisfied, people will ask for a higher quality of life, such as a better environment [34,35,36]. Demographic factors are people’s inherent attributes that are supposed to exert influences on people’s environmentally significant behaviours. However, few studies have been conducted to analyse this association within the social background of rural China. Based on the above research backboard, this paper seeks to examine this association by verifying the following hypotheses: females, younger generations, and people with higher education and income are more likely to form environmentally motivated behaviours than males, the elderly, and those who have lower education and income.

## 4. Method

### 4.1. Participants and Procedure

A survey was conducted in 2014 in Ningyang county which is located in the middle of Shandong province of China. In 2014, the census registered population in Ningyang was 830,000, including 629,000 agricultural households and 202,000 non-agricultural households. The urban per capita disposable income in 2014 was CNY25,427, and rural per capita net income was CNY12,010. Multistage sampling was adopted to select the samples in Ningyang. First, a list including all 13 township level districts was prepared. Taking the first district as the starting point, seven towns were selected at an equal interval from the list. The selected seven towns have 315 villages and 370,188 residents in total. Second, 51 villages were proportionately selected based on the population in each village. Finally, based on the designated gender and age categories, 10 individuals in each selected village were chosen. Ten students from the local area were recruited and trained as the interviewers. They were asked to finish five face to face interviews per day. The start and end time of each interview were recorded on the front page of the questionnaires. On average, one interview lasted approximately 30 min.

In total, 508 valid samples were successfully collected. Overall, the samples represented gender and age categories well. Half of the respondents were males (*n* = 254), and half were females. Samples were distributed on average in the designated age categories, 18–29 years (*n* = 103), 30–39 years (*n* = 99), 40–49 years (*n* = 102), 50–59 years (*n* = 102), and 60 years and over (*n* = 102).

### 4.2. Measurement

In the survey, the respondents were asked how often they performed the selected five behaviours in their daily life, including the purchase of eco-friendly products, reuse or recycling, water saving, energy saving, and the use of their own shopping bag. The scale included “do so always”, “sometimes”, “not very often”, and “not at all”. If the respondents chose the first and second options, they were further asked the reason why they did so. Two types of motivations, economic motivations and environmental motivations, were measured by the description “to save money” and “in consideration of the environment”, respectively. The specific question items are shown in Table 1.

People’s environmental anxiety was assessed by asking “to what extent do you worry, either for yourself or for your family, about environmental deterioration?” The scale items included “very much”, “somewhat”, “slightly”, and “not at all”. The ascription of environmental responsibility was assessed by asking “among the government, corporations, and citizens, who do you think should play the most important role in protecting the environment?” Demographic factors, including gender, age, educational level, and yearly household income were also interviewed.

In the analysis, age, education, and yearly household income were divided into three categories as follows: (1) age factor: young age, including those aged 18–34 years (27%); middle age, including those aged 35–49 years (45.5%); and old age, including those aged 50 years and over (27.6%); (2) education factor: low education, including less than one year’s study and elementary school (37.8%); middle education, including junior high school (39.4%); and high education, including high school, junior college, vocational school, university, and graduate school (22.9%); (3) income factor: low income, including less than 20,000 yuan (39.2%); middle income, including 20,000 yuan to less 50,000 yuan (47%); and high income, including 50,000 yuan and over (13.8%).

### 4.3. Analytic Approach

The relative frequencies of the questions were first checked to grasp the response features of the surveyed rural areas. A proportion test was then supplemented to clarify whether there was a significant difference in performing environmentally significant behaviours in rural and urban China. The results report not only the statistical significance (*p* value) but also the substantive significance (effect size). The index used in this study is Cohen’s d value, which is classified as small (*d* = 0.2), medium (*d* = 0.5), and large (*d* = 0.8). To explore the formation mechanism of environmentally motivated behaviours, a multiple correspondence analysis (MCA) and logistic regression modelling were conducted. The MCA was used to clarify the general patterns of the relationships between the motivations and the proposed influencing factors. A logistic regression modelling was conducted to specify the influencing degree of each influencing factor. Regression coefficients and *p* values are provided.

## 5. Results

### 5.1. Practice of Environmentally Significant Behaviours in Surveyed Rural Areas

In the survey, the respondents were asked how often they have performed the proposed five environmentally significant behaviours in the past year in their daily life, and their responses are shown in Table 2.

As shown in Table 2, the author found that approximately 80% of the respondents indicated that they were doing the surveyed behaviours “always” or “sometimes”, except using their own shopping bag. Regarding the purchase of eco-friendly products, 79.2% of respondents indicated they were doing so “always” or “sometimes”. Regarding the behaviour of reuse or recycling, 87.5% of respondents indicated that they were doing so “always” or “sometimes”. Regarding the behaviours of “water saving” and “energy saving”, approximately 90% of the respondents indicated that they were doing so “always” and “sometimes”, and it is noted that approximately 50% of the respondents indicated that they were doing these behaviours “always” in their daily life. Regarding the use of their own shopping bag, only 45.7% of the respondents indicated that they were doing so “always” or “sometimes”.

Based on the above results, it can be seen that, except in the use of one’s own shopping bag, the practical rate of the surveyed behaviours in rural areas is relatively high. The main motivation that drives people to do these behaviours was also investigated. Two options, “to save money” and “in consideration of the environment”, were provided. The responses to these subsequent questions are shown in Table 2.

As shown in Table 2, the author found that a considerable proportion of the rural residents were doing these behaviours to save money instead of considering the environment. Only 33.9% of the respondents who were reusing or recycling, 30.4% of the respondents who were saving water, and 20.5% of the respondents who were saving energy indicated that they were doing these behaviours because of the “environment”, whereas the majority of people were doing so to “save money”. Regarding the reason for purchasing eco-friendly products and using their own shopping bag, more than half of the respondents indicated they were doing so “in consideration of the environment”.

To provide a reference to evaluate the above practice of environmentally significant behaviours in rural areas, previous data [7,37,38] collected from two cities, Beijing located in northern China and Hangzhou located in southern China, were introduced and compared. The author also attended the survey in these cities. The same survey method (face-to-face interview), the same survey questions (see Table 1), and a similar sampling method (multistage sampling) (see detailed information in Appendix A) made this comparison possible. The comparative results are also shown in Table 2.

Based on the proportion test, the author found that compared to the urban areas of China, the practice of the surveyed behaviours in rural areas is not necessarily lower. Regarding reuse and recycle, the percentage of “always” or “sometimes” in rural areas was lower than that in Beijing; however, it was significantly higher than that in Hangzhou. Regarding water saving, the percentage of “always” or “sometimes” in rural areas was significantly lower than that in Beijing; however, it was significantly higher than that in Hangzhou. Regarding energy saving, the percentage of “always” or “sometimes” in rural areas was significantly lower than that in Beijing; however, it was still higher than that in Hangzhou.

However, in considering the motivation that underlies these behaviours, there was a consistent tendency that the percentages of “in consideration of the environment” of all behaviours in rural areas were lower than that in both Beijing and Hangzhou. It is noted that these differences between rural areas and these two cities were all proved to be significant except the purchasing of eco-friendly products. Therefore, it can be said that although the practice of environmentally significant behaviours in rural areas is not necessarily lower than that in urban areas, these behaviours are definitely more economically motivated rather than environmentally motivated.

### 5.2. Causal Analysis of Factors Leading to Environmentally Motivated Behaviours

Environmentally motivated behaviours are supposed be more reliable and durable than economically motivated behaviours. The following analyses were focused on clarifying the influencing factors that affect the formation of environmentally motivated behaviours in rural areas. An MCA was first conducted to visually display the mutual relationship among the motivations, AC, AR, and demographic factors. Logistic regression modelling was then performed to clarify the degree to which the proposed factors influence the formation of environmentally motivated behaviours. Individuals who answered that they were doing the surveyed behaviours “always” or “sometimes” were included in the analyses. Responses to AC and AR question items were shown in Appendix B.

#### 5.2.1. MCA of the Relationship among Motivations, AC, AR, and Demographic Factors

**Figure 1:**
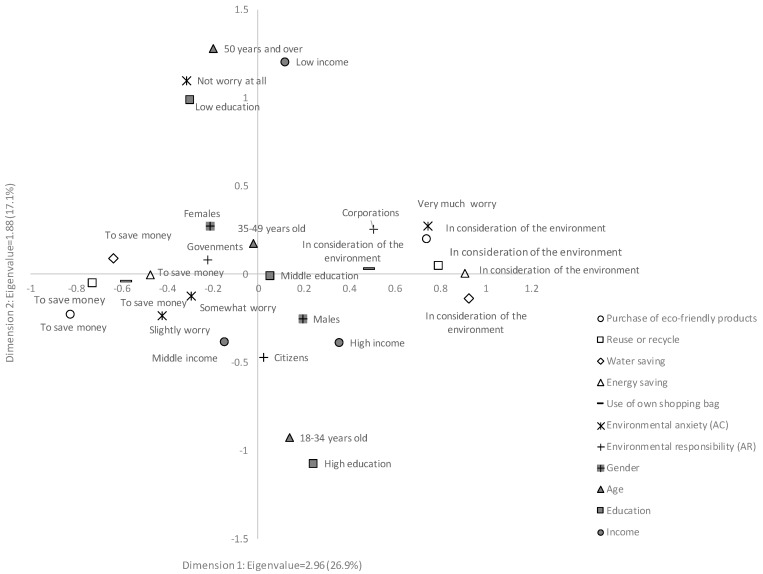
Multiple correspondence analysis of the relationship among motivations, AC, AR, and demographic factors.

The spatial pattern classification in this method was demonstrated as a two-dimensional configuration in Figure 1.

The two largest eigenvalues were 2.96 (contribution: 26.9%) and 1.88 (contribution: 17.1%). If two categories in the coordinate are near each other, we can say that the relationship between them is close. Based on dimension 1, people’s motivations are clearly divided into two groups. The motivation of “in consideration of the environment” and the options of “very much worried”, “corporation responsibility”, and “male” are located in the positive direction of dimension 1; the motivation of “to save money” and the options of “slightly and somewhat worried”, “government responsibility”, and “female” are located in the negative direction of dimension 1. The other options are generally located along dimension 2. Based on this spatial pattern in Figure 1, the following tendencies in rural areas were indicated: (1) people with more environmental anxiety are more likely to be motivated by environmental considerations, whereas less anxiety is associated with money saving; (2) people who ascribed the most important environmental responsibility to corporations are more likely to be motivated by environmental considerations, whereas people who ascribe it to government are more likely to consider money saving; and (3) compared to females in rural areas, males are more likely to be motivated by environmental considerations.

According to the MCA, the relationship among people’s behaviour motivations, AC, AR, and demographic factors were clarified, and furthermore, some important tendencies regarding the influences of the proposed factors were derived from the analysis. To specify the magnitude of each influencing factor and to determine whether these influences were statistically significant, the following logistic regression modeling was conducted.

#### 5.2.2. Logistic Regression Modelling of the Causal Effects of AC, AR, and Demographic Factors

Motivations (in consideration of the environment = 1, to save money = 0) were set as the dependent variable, AC, AR, and four demographic factors including gender, age, education, and income were set as the independent variables, and a binary logistic regression analysis was conducted. In the analysis, the options of “not worry” (including slightly and not at all), “government responsibility”, “male”, “old age”, “low education”, and “low income” were set as the reference categories. The analysis results are shown in Table 3 (see odds ratio and its 95% confidence interval in Appendix C).

Regarding the behaviour of purchasing eco-friendly products, the significant influences of AC, AR, and age were confirmed. First, people’s environmental anxiety affected behavioural motivations significantly and positively (*β* = 0.96, *p* ≤ 0.001). The more people worry about the environment, the more likely they will be to purchase eco-friendly products due to environmental motivation. Second, people who ascribed the most important environmental responsibility to corporations (*β* = 0.496, *p* ≤ 0.1) were more likely to purchase eco-friendly products due to environmental motivation than those who ascribed it to the government. Third, compared to the old, middle aged people (*β* = 0.96, *p* ≤ 0.001) were more likely to purchase eco-friendly products due to environmental motivation.

Regarding the behaviour of reuse or recycling, the significant influences of AC, AR, gender, and age were confirmed. First, people’s environmental anxiety affected behavioural motivations significantly and positively (*β* = 1.075, *p* ≤ 0.001). The more people worry about the environment, the more likely they will be to reuse or recycle due to environmental motivation. Second, people who ascribed the most important environmental responsibility to citizens (*β* = 0.571, *p* ≤ 0.05) were more likely to reuse or recycle due to environmental motivation than those who ascribed it to the government. Third, compared to males, females (*β* = −0.623, *p* ≤ 0.01) were less likely to reuse or recycle due to environmental motivation. Fourth, compared to the elderly, young people (*β* = 1.142, *p* ≤ 0.001) and middle aged people (*β* = 0.59, *p* ≤ 0.1) were more likely to reuse or recycle due to environmental motivation.

Regarding the behaviour of water saving, the significant influences of AC and income were confirmed. First, people’s environmental anxiety affected behavioural motivations significantly and positively (*β* = 1.255, *p* ≤ 0.001). The more people worry about the environment, the more likely they will be to save water due to environmental motivation. Second, compared to the people who have low income, people with high income (*β* = 0.836, *p* ≤ 0.05) and middle income (*β* = 0.561, *p* ≤ 0.05) were likely to save water due to environmental motivation.

Regarding the behaviour of energy saving, the significant influences of AC, AR, and education were confirmed. First, people’s environmental anxiety affected behavioural motivations significantly and positively (*β* = 1.181, *p* ≤ 0.001). The more people worry about the environment, the more likely they will save energy due to environmental motivation. Second, people who ascribed the most important environmental responsibility to citizens (*β* = 0.687, *p* ≤ 0.05) were more likely to save energy due to environmental motivation than those who ascribed it to the government. Third, compared to the people who have low education, people with middle education (*β* = 0.752, *p* ≤ 0.05) were likely to save energy due to environmental motivation.

Finally, regarding the behaviour of using one’s own shopping bag, the significant influence of AR was confirmed. People who ascribed the most important environmental responsibility to corporations (*β* = 0.769, *p* ≤ 0.1) were more likely to use their own shopping bag due to environmental motivation than those who ascribed it to the government.

Based on the above results, the determinants and their influencing magnitude of motivations were examined. Overall, the influence of environmental anxiety is strong and stable. The more people worry about the environment, the more likely they are to form environmentally motivated behaviours. Although not significant for all analysed behaviours, the governmental responsibility ascription generally leads to economically motivated behaviours. People who ascribed the most important environmental responsibility to citizens or corporations were more likely to form environmentally motivated behaviours. The hypotheses on environmental anxiety and responsibility were generally verified. Regarding the influence of gender, in contrast to the hypothesis, males are generally more likely to have environmentally motivated reuse or recycle behaviours. Younger generations including the young and middle-aged people were more likely to purchase eco-friendly products, or to reuse or recycle, because of environmental motivation. Finally, people with high and middle income and middle education were more likely to have environmentally motivated water and energy saving behaviours. The hypotheses on the influence of income and education were verified on some of the environmentally significant behaviours.

## 6. Discussion

As one of the few studies that focuses on environmentalism in rural areas of China, this paper provides some descriptive information regarding rural residents’ environmentally significant behaviours in present-day China. Five environmentally significant behaviours, including the purchase of eco-friendly products, reuse or recycling, water saving, energy saving, and the use of one’s own shopping bag, were investigated. Analytical results derived from the survey data revealed that these environmentally significant behaviours were widely conducted in rural life, which are beneficial and necessary for rural sustainability. However, it was noted that some features were indicated on some of the behaviours. First, the practical rate of using one’s own shopping bag was much lower compared to other environmentally significant behaviours, and it was also significantly lower than that in referenced cities. One possible reason may be the implementation of free plastic bags ban that came into effect on 1 June 2008 in China. This ban asks the stores to charge the consumers for plastic bags, which have substantially reduced the use of plastic bags. However, the implementation of the ban in rural areas is much looser, and consumers can still obtain free plastic bags from the stores. Second, the purchase of eco-friendly products and the use of one’s own shopping bag were more likely to be motivated by environmental motivation than other environmentally significant behaviours. This result may indicate a different attribute of these two behaviours. Although they were discussed as “private sphere” behaviours in this paper, they can be very “public facing” behaviours. People who engage in these types of behaviors may not do so for environmentalism as they claim, but to be seen to be doing so.

In addition, two motivations underlying the behaviours, including environmental motivation and economic motivation, were examined. Statistical analysis of the surveyed data indicated that although environmentally significant behaviours are widely conducted in rural areas, these behaviours are mainly motivated by economic gains rather than environmental considerations. This, to a large extent, is determined by the less developed socioeconomic situation in rural areas. The reason can also be derived from the Maslow’s hierarchy of needs [34] or Inglehart’s materialist and post-materialist theory [35]. Compared to the cities, rural areas of China generally have lower mean income, a lower standard of living, and a lack of provision of social infrastructure. In such a socioeconomic context, it is not surprising that people have a strong tie to the economic system and adopt behaviours dominated by economic motivation. “Survival has always depended on the careful stewardship of finite resources” [21]. However, it should be noted that frugality is always a praiseworthy virtue in China, especially in rural China. Environmentally significant behaviours motivated by economic motivations should also be advocated in present-day China because they make a continuous difference to the rural environment.

However, from a long-term perspective, it is of particular importance to make an effort to achieve a shift from being motivated by economic gains to being motivated by environmental considerations. Analytical results derived from the survey data in this paper supplied some clues to realize this shift.

First, increasing people’s anxiety towards environmental deterioration is helpful in influencing environmentally motivated behaviours. This positive relationship between AC and various environmentally significant behaviours has been verified in many previous research [23,26,39,40]. However, there are rare previous references as to how to increase people’s AC. In the presently studied rural areas, more access to environmental information via TV or diverse rural activities may be an effective way to increase people’s worries about environmental deterioration. Rural areas of China are generally enclosed communities [41]. The poor exchange of environmental information may prevent people from enacting environmentally significantly behaviours. In the long-term, systemic research should focus on examining the formation mechanism of rural residents’ environmental consciousness, which is deemed as the fundamental element that evokes people’s environmentally significantly behaviours in daily life.

Second, decreasing people’s dependency on the government in protecting the environment is beneficial in promoting environmentally motivated behaviours. In the surveyed rural areas, approximately half of the respondents ascribed the most important environmental responsibility to the government. However, analytical results in this paper indicated that the ascription of environmental responsibility to the government leads to less environmentally motived behaviours. The poorer economic base and lower social development in rural society may make rural residents lack confidence in their ability to change the environment, and therefore, they turn to the government to find the solution. More efforts should be dedicated to invoking rural residents’ responsibility and the efficacy of themselves and other social actors in protecting the environment. Making rural residents aware that many of their behaviours in daily life can not only bring them economic interest but can also help the environment substantially may be an effective way to achieve this goal.

Third, females, the elderly, and people with low income and education levels should be targeted for environmental education in rural China. Considering the features of the targeted populations, educational measures should be easy to access and understand, and they cost less. Contrary to the common conclusion in previous research, females in rural areas were less likely to be environmentally motivated than males. Possible reasons may be that males in rural areas are more involved in public issues of communities and are open to environmental information, whereas females mainly engage in housework. In recent years, many male peasant-workers have been working in cities and leaving their wives at home to take care of the families and farming. City work may make the males become more environmentally concerned. However, since females play the main role in daily housework, it is thus particularly important to realize the motivational shift of females’ environmentally significant behaviours.

However, there are several limitations that need to be addressed: first, results are based largely on single-variable assessments of environmental anxiety, the ascription of environmental responsibility, and each environmentally significant behaviour. More systematic measurements of these variables are needed; second, environmental anxiety and the ascription of environmental responsibility analysed in this paper are loose adaptations of the AC and AR of norm-activation theory. More precise measurement and application of these factors are expected to supply more information regarding how to improve people’s environmentally significant behaviours.

Rural areas of China are now facing increasingly serious environmental issues. This study supplied primary data regarding the status of environmentally significant behaviours in rural China, and more importantly, identified some influencing factors that lead to environmentally motivated behaviours. However, this is a preliminary exploration, and further quantitative analyses and discussion on environmental consciousness and behaviours in rural China are urgently needed to provide more detail of and effective solutions to rural environmental degradation.

## Figures and Tables

**Table 1:** Question items regarding environmentally significant behaviours and motivations.

Item Name	Question		
Purchase of eco-friendly products	We are now going to show you a list of several activities that you could be doing at the level of daily life. How often have you performed each of them during the past year or so? Please choose one that comes closest to your actions. (Note to interviewers: For each item from A to E, ask the follow up “SQ” question if the respondent has selected 1 or 2.)	A. Buy products that are energy-efficient and/or have been designated by government as eco-friendly.	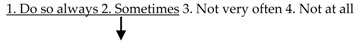
		SQ. What is your reason for doing so?	1. To save money 2. In consideration of the environment
Reuse or recycle		B. Recycle things, or otherwise avoid throwing them away so as to reuse them again.	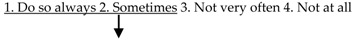
		SQ. What is your reason for doing so?	1. To save money 2. In consideration of the environment
Water saving		C. Try to avoid overusing water in washing things or in the shower.	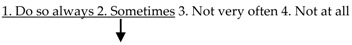
		SQ. What is your reason for doing so?	1. To save money 2. In consideration of the environment
Energy saving		D. Try to use energy for lighting, heat or air conditioning and so on, in moderation.	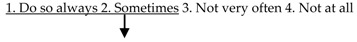
		SQ. What is your reason for doing so?	1. To save money 2. In consideration of the environment
Use of own shopping bag		E. Turn down offers for bags or packaging during shopping and use your own shopping bag.	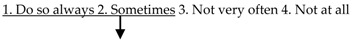
		SQ. What is your reason for doing so?	1. To save money 2. In consideration of the environment

**Table 2:** Relative frequencies and proportion test results of responses to behaviours and motivations items (Unit: %).

		Rural Areas		Urban Areas							
			Ningyang	Beijing	Difference	*p*-Value	*d*-Value	Hangzhou	Difference	*p*-Value	*d*-Value
BEHAVIOURS	Purchase of eco-friendly products	Do so always	23.5	44.7	11.5	***	**	31.4	1.8		
		Sometimes	55.7	46.0				49.6			
		Not very often	20.2	6.4	−11.5	***	**	17.7	−1.9		
		Not at all	0.6	2.9				1.2			
	Reuse or recycle	Do so always	31.7	41.6	0.8			22.7	−8.9	***	*
		Sometimes	55.8	46.7				55.9			
		Not very often	12.3	9.2	−0.8			20.5	8.9	***	*
		Not at all	0.2	2.5				0.9			
	Water saving	Do so always	49.7	73.8	3.6	*	*	47.4	−5.8	**	*
		Sometimes	40.8	20.3				37.3			
		Not very often	9.1	5.1	1.4	*	*	14.5	5.0	**	*
		Not at all	0.4	0.8				0.8			
	Energy saving	Do so always	53.7	71.7	4.7	**	*	48.3	−3.4		
		Sometimes	35.8	22.5				37.8			
		Not very often	10.6	5.1	−4.9	**	*	13.6	3.4		
		Not at all	0.0	0.6				0.4			
	Use of own shopping bag	Do so always	16.5	60.2	43.5	***	***	47.2	33.7	***	***
		Sometimes	29.2	29.0				32.2			
		Not very often	46.1	7.5	−43.5	***	***	18.3	−33.7	***	***
		Not at all	8.2	3.3				2.3			
MOTIVATIONS	Purchase of eco-friendly products	To save money	47.7	30.1	−17.6	***	*	42.8	−4.9		
		In consideration of the environment	52.3	69.9	17.6	***	*	57.2	4.9		
	Reuse or recycle	To save money	66.1	38.3	−27.8	***	**	57.0	−9.1	**	*
		In consideration of the environment	33.9	61.7	27.8	***	**	43.0	9.1	**	*
	Water saving	To save money	69.6	36.9	−32.7	***	**	56.0	−13.6	***	*
		In consideration of the environment	30.4	63.1	32.7	***	**	44.0	13.6	***	*
	Energy saving	To save money	79.5	50.8	−28.7	***	**	69.3	−10.2	***	*
		In consideration of the environment	20.5	49.2	28.7	***	**	30.7	10.2	***	*
	Use of own shopping bag	To save money	46.9	26.2	−20.7	***	**	40.0	−6.9		
		In consideration of the environment	53.1	73.8	20.7	***	**	60.0	6.9		

Note: 1. This table summarizes the relative frequencies of interviewees’ responses to the behaviours and motivations question items. To provide a reference, previous data collected from urban areas were provided. 2. “Difference” refers to the percentage differences between Ningyang and Beijing/Hangzhou and was calculated by using Beijing/Hangzhou percentages minus Ningyang percentages. 3. The proportion test was conducted to confirm whether the “difference” was significant on the statistics. *p* values: *p* ≤ 0.1, * *p* ≤ 0.05, ** *p* ≤ 0.01, *** *p* ≤ 0.001; *d* values: * *d* ≥ 0.2, ** *d* ≥ 0.5, *** *d* ≥ 0.8.

**Table 3:** Logistic regression analysis of causal effects of AC, AR, and demographic factors (coefficient *β* and *p* value).

		Purchase of Eco-Friendly Products	*p*-Value	Reuse or Recycle	*p*-Value	Water Saving	*p*-Value	Energy Saving	*p*-Value	Use of Own Shopping Bag	*p*-Value
	Intercept	−1.048	**	−2.175	***	−2.427	***	−2.985	***	−0.882	
AC & AR	Worry [vs. Not worry]	0.960	***	1.075	***	1.255	***	1.181	***	0.444	
	Citizens [vs. Governments]	0.234		0.571	*	0.157		0.687	*	0.259	
	Corporations [vs. Governments]	0.496		0.305		0.241		0.275		0.769	
Demographic factors	Female [vs. Male]	−0.072		−0.623	**	−0.136		−0.045		0.056	
	18–34 years [vs. 50 years and over]	0.242		1.142	**	0.161		0.227		−0.158	
	35–49 years [vs. 50 years and over]	0.646	*	0.590		−0.127		−0.107		−0.312	
	High education [vs. Low education]	0.281		0.320		0.453		0.624		0.794	
	Middle education [vs. Low education]	−0.002		0.358		0.197		0.752	*	0.280	
	High income [vs. Low income]	−0.222		0.462		0.836	*	0.386		0.109	
	Middle income [vs. Low income]	−0.320		−0.074		0.561	*	−0.219		0.421	

Note: 1. *p*-value: *** *p* ≤ 0.001, ** *p* ≤ 0.01, * *p* ≤ 0.05, *p* ≤ 0.1. 2. coefficients *β* > 0 represents a positive effect on environmental motivation, which indicants that people with such a feature are more likely to have environmentally motivated behaviours, whereas *β* < 0 represents a negative effect on environmental motivation, which indicants that people with such a feature are more likely to have economically motivated behaviours.

## Appendix A

Survey and sampling information in Beijing and Hangzhou

1. Survey time: 2011
  (1) Based on the population of the districts, 100 communities were selected
  (2) Based on the population of designated gender and age categories, 10 individuals were selected in each community.
2. Sampling method: Multistage sampling (quota)
3. Survey method: Face-to-face interview
4. Sample size: Beijing, 1000 persons; Hangzhou, 1011 persons.

## Appendix B

**Table A1:** Responses to environmental anxiety and environmental responsibility question items.

Factor	Options	(%)
Environmental anxiety (AC)	1. Very much	18.6
	2. Somewhat	48.1
	3. Slightly	28.3
	4. Not at all	5.0
Environmental responsibility (AR)	1. Government	48.9
	2. Corporation	25.2
	3. Citizen	26.0

## Appendix C

**Table A2:** Logistic regression analysis of causal effects of AC, AR, and demographic factors (odds ratio and its 95% confidence interval).

		Purchase of Eco-Friendly Products			Reuse or Recycle			Water Saving			Energy Saving			Use of Own Shopping Bag		
		Odds ratio	95% C.I.		Odds ratio	95% C.I.		Odds ratio	95% C.I.		Odds ratio	95% C.I.		Odds ratio	95% C.I.	
	Intercept	0.351	[0.170	0.725]	0.114	[0.054	0.237]	0.088	[0.040	0.194]	0.051	[0.020	0.128]	0.414	[0.157	1.094]
AC & AR	Worry [vs. Not worry]	2.612	[1.551	4.400]	2.929	[1.719	4.990]	3.509	[1.949	6.316]	3.258	[1.630	6.511]	1.558	[0.740	3.283]
	Citizens [vs. Governments]	1.264	[0.712	2.242]	1.769	[1.009	3.101]	1.170	[0.657	2.086]	1.988	[1.050	3.761]	1.295	[0.605	2.774]
	Corporations [vs. Governments]	1.642	[0.959	2.809]	1.357	[0.787	2.341]	1.273	[0.741	2.186]	1.316	[0.702	2.468]	2.158	[0.990	4.706]
Demographic factors	Gender [Female]	0.930	[0.587	1.474]	0.536	[0.338	0.850]	0.873	[0.549	1.388]	0.956	[0.562	1.625]	1.057	[0.559	2.000]
	Age [18–34 years]	1.273	[0.600	2.703]	3.133	[1.480	6.634]	1.175	[0.561	2.463]	1.255	[0.538	2.928]	0.854	[0.294	2.480]
	Age [35–49 years]	1.908	[1.030	3.536]	1.804	[0.951	3.423]	0.881	[0.472	1.644]	0.899	[0.430	1.878]	0.732	[0.306	1.751]
	Education [High education]	1.324	[0.653	2.682]	1.377	[0.685	2.768]	1.574	[0.781	3.172]	1.866	[0.812	4.291]	2.213	[0.857	5.714]
	Education [Middle education]	0.998	[0.564	1.766]	1.431	[0.799	2.560]	1.218	[0.678	2.190]	2.122	[1.054	4.272]	1.323	[0.621	2.816]
	Income [High income]	0.801	[0.398	1.615]	1.586	[0.781	3.221]	2.307	[1.136	4.688]	1.471	[0.684	3.164]	1.115	[0.398	3.122]
	Income [Middle income]	0.726	[0.427	1.234]	0.929	[0.549	1.571]	1.753	[1.017	3.020]	0.804	[0.434	1.488]	1.524	[0.700	3.320]
